# Insight into the LFA-1/SARS-CoV-2 Orf7a Complex by Protein–Protein Docking, Molecular Dynamics, and MM-GBSA Calculations

**DOI:** 10.1021/acs.jcim.1c00198

**Published:** 2021-05-27

**Authors:** Alberto Ongaro, Erika Oselladore, Maurizio Memo, Giovanni Ribaudo, Alessandra Gianoncelli

**Affiliations:** Department of Molecular and Translational Medicine, University of Brescia, Viale Europa 11, 25123 Brescia, Italy

## Abstract

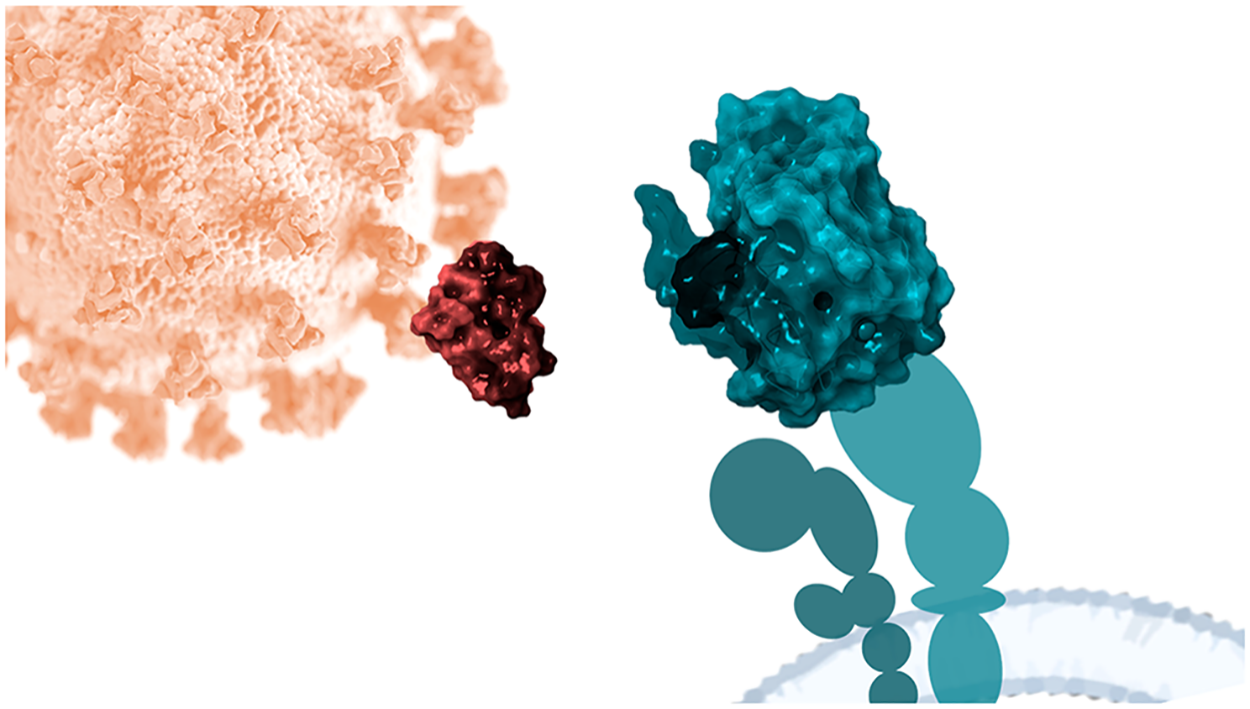

In the Severe Acute Respiratory Syndrome Coronavirus 2 (SARS-CoV-2) genome, open reading frames (ORFs) encode for viral accessory proteins. Among these, Orf7a structurally resembles the members of the immunoglobulin (Ig) superfamily and intracellular adhesion molecules (ICAMs), in particular. ICAMs are involved in integrin binding through lymphocyte function-associated antigen 1 (LFA-1). Based on such considerations and on previous findings on SARS-CoV, it has been postulated that the formation of the LFA-1/Orf7a complex could contribute to SARS-CoV-2 infectivity and pathogenicity. With the current work, we aim at providing insight into this macromolecular assembly, taking advantage of the recently reported SARS-CoV-2 Orf7a structure. Protein–protein docking, molecular dynamics (MD) simulations, and a Molecular Mechanical-Generalized Born Surface Area (MM-GBSA)-based stage were enrolled to provide refined models.

## Introduction

The genome of coronaviruses consists of a positive-stranded RNA sequence, which encodes for some specific viral components such as replicase, spike, envelop, and nucleocapsid proteins. Additionally, open reading frames (ORFs) encode for accessory proteins which are not crucial for viral replication but may be relevant for virus–host interactions, infectivity, and pathogenicity.^1^ Among these, ORF7A encodes for accessory protein 7a (Orf7a), which was previously reported to be expressed in Severe Acute Respiratory Syndrome Coronavirus (SARS-CoV)-infected cells, both in the endoplasmic reticulum (ER) compartment and on the cell surface.^2−5^ Moreover, Huang et al. described its presence in viral particles of SARS-CoV,^6^ and coimmunoprecipitation assays demonstrated that Orf7a interacts with 3a and the spike protein in virions.^7^

Nelson et al. solved the structure of the SARS-CoV Orf7a, highlighting the presence of a seven-stranded β-sandwich resembling in fold and topology the one found in intracellular adhesion molecule-2 (ICAM-2), a member of the immunoglobulin (Ig) superfamily.^5^ ICAMs are cell adhesion molecules and are specialized in integrin binding: ICAM-1 and ICAM-2 specifically interact with lymphocyte function-associated antigen 1 (LFA-1), which is mainly expressed on lymphocytes. Although sequence identity between SARS-CoV Orf7a and ICAM-1 and ICAM-2 is limited, the three-dimensional structures are very similar. Furthermore, both ICAMs share with SARS-CoV Orf7a the Glu residues (Glu37 and Glu26, respectively) and the hydrophobic surrounding ring, which are crucial for the interaction with LFA-1.^1^ LFA-1 mediates adhesive interactions among cells of the immune system, and more in general, integrins are deputed to mediating cell–cell interactions and regulating cell-matrix adhesion.^8^ The LFA-1/ICAM interaction pattern is characterized by the presence of a flat surface at the interface involving a metal ion-dependent binding site (MIDAS) (PDB ID: 1MQ8).^9^ Starting from these coordinates, Hänel et al. proposed a structural model for the LFA-1/Orf7a complex, which was predicted *in silico* using structure alignment.^1^ Further studies experimentally confirmed that SARS-CoV Orf7a and LFA-1 interact *in vitro*, supporting the hypothesis that LFA-1 could be an attachment factor or the receptor for SARS-CoV on human leukocytes.^10^ Preliminary computational studies suggest that SARS-CoV-2 could show the same behavior.^11^ In this connection, Tan et al. previously demonstrated that, similarly to what was observed in other infections from coronaviruses, Orf7a from SARS-CoV induces apoptosis mediated by a caspase-dependent pathway in cell lines derived from lung, kidney, and liver.^7^

The possible biological outcomes of the interaction of SARS-CoV or SARS-CoV-2 Orf7a with LFA-1 clearly depend on the localization of this accessory protein: in this connection, three main theories arise.^10^ (I) The presence of Orf7a on the virus surface would enable using LFA-1 for cell entry, similar to a mechanism observed for HIV.^12^ Huang et al. reported the localization of Orf7a in viral particles of SARS-CoV,^6^ thus LFA-1 could represent an attachment factor or a receptor for the virus.^10^ This would help in justifying the fact that SARS-CoV infects cells which do not express ACE2, such as T cells.^13^ (II) ER localization suggests that Orf7a may block LFA-1 molecules’ transition from ER to cell surface. As LFA-1 is expressed in leukocytes, loss of LFA-1 could cause defects of the immune system.^14^ (III) The presence of Orf7a on the surface of infected cells suggests interference with T cell homing and increased affinity of such cells for leukocytes, eventually inducing caspase-dependent apoptosis in LFA-1-expressing T cells.^3,5^ Lymphopenia was indeed reported for SARS patients.^15^

Viral proteins bearing Ig-like domains are currently captivating attention, as demonstrated by recent reports on SARS-CoV-2.^16^ Further investigations are needed to assess if Orf7a could be considered among the potential druggable targets to contrast SARS-CoV-2,^17,18^ and ORF7a gene mutations/deletions are being evaluated to distinguish genomic populations.^19,20^ Moreover, the involvement of Orf7a in interfering with human immune response has been recently hypothesized,^21^ and growing evidence supports its role in COVID-19 pathogenesis.^22^

The current study aims at providing structural insight into the LFA-1/SARS-CoV-2 Orf7a complex by means of computational methods, taking advantage of the SARS-CoV-2 Orf7a structure that was recently reported (PDB ID: 6W37).^23^ The poses resulting from protein structure alignment and protein–protein docking experiments were subjected to molecular dynamics (MD) simulations to evaluate their stability over time in a simulated aqueous environment. A further free energy calculation study based on the Molecular Mechanical-Generalized Born Surface Area (MM-GBSA) approach allowed for highlighting the most stable models showing the lowest energy values. Eventually, the predicted binding patterns were compared to that of efalizumab, a monoclonal antibody targeting LFA-1.

## Materials and Methods

### Generation of LFA-1/Orf7a Complex Models

The structures for LFA-1, Orf7a, and for the LFA-1/ICAM complex were retrieved from the Protein Data Bank (PDB). For LFA-1, the 3F74(24) crystal at 1.7 Å resolution was selected. For SARS-CoV-2 Orf7a, crystal 6W37(23) at 2.90 Å resolution was chosen, whereas for SARS-CoV Orf7a, the solution NMR structure 1YO4(1) was selected. For the LFA-1/ICAM complex, 1MQ8(9) and 1T0P(25) crystals, respectively, at the resolution of 3.3 and 1.65 Å, were used.

All the protein structures were loaded as PDB files in Schrödinger 2020 and prepared with the embedded Protein Preparation Wizard^26^ application using default settings, as reported in a previous work,^27^ i.e., adding hydrogens, assigning disulfide bonds, removing surrounding waters, adjusting charges, capping termini, and adding missing side chains using Prime.^28^ The optimization of hydrogen bonds was performed to resolve structural ambiguities, and a final restrained minimization of the system was carried out under the OPLS3e force field. In greater detail, a full optimization for hydrogen atoms and a 0.30 Å maximum RMSD deviation from the initial position for the heavy atoms were allowed.^29^

For sequence and structural alignment studies, the structures were superimposed using Multiple Sequence Viewer and Protein Structure Alignment applications embedded in the Schrödinger suite. The first one gives an identity percentage aligning the residues, whereas the latter one provides an RSMD value, calculated on the C-alpha atoms of the aligned residues, and an “Alignment Score”, which introduces a protein structure distance (PSD) term, designed to include a quantitative measure of structural similarity through equivalent secondary structural element, particularly useful when the proteins share less sequence identity. The score is calculated so that it approaches zero when the two proteins are identical, while it increases when the two proteins differ from each other.^30^

The protein–protein docking experiments were performed using the HDOCK server (http://hdock.phys.hust.edu.cn/),^31^ which is based on a hybrid algorithm of template-based modeling and *ab initio* free docking. In greater detail, the scoring function adopted by HDOCK was obtained by improving the widely used Fast-Fourier Transform (FFT)-based algorithm with a long-range shape-based scoring (LSC) function. During sampling, the score for a ligand grid takes into account the contributions not only from the nearest neighboring receptor grids but also from other receptor grids, depending on distance parameter *r* (by the form of ∼*e*^–1/*r*^2^^). The ligand is rotated and translated: the top 10 translations for each rotation are optimized by the iterative knowledge-based scoring function, which is able to predict the reference state and therefore allows the extraction of realistic interaction potentials. This results in one binding mode for each rotation. The binding modes are then clustered with an RMSD cutoff of 5 Å, where the RMSD is calculated using backbone atoms.^32−34^ The PDB structures of LFA-1and Orf7a/ICAM were uploaded to the HDOCK server as receptor and ligands, respectively, using default settings. After the docking calculations, the result files with the docking scores and the docked structures were retrieved from the server. The docked structures, in PDB format, were directly loaded on Schrödinger 2020^35^ for structure inspection and for the following computational studies.

### MD Simulations

The MD experiments were carried out using Desmond 2020^36^ installed on a Linux machine. The simulations were performed through GPU acceleration on Nvidia CUDA. All the protein structures, referred to as “models” in the following, were prepared with the Protein Preparation Wizard application embedded in Schrödinger 2020 following the procedure described before for the protein–protein docking. The System Builder application embedded in Desmond was used to prepare the systems for the subsequent calculations. The TIP3P water model was used to solvate the proteins enclosed in an orthorhombic cage with a 10 Å buffer area. Na^+^ ions were added to neutralize the system, and a concentration of 0.15 M of NaCl was simulated. Before submitting MD simulations, the systems were equilibrated with the default relaxation protocol of Desmond, which includes two stages of minimization (restrained and unrestrained) followed by four stages of MD runs with gradually diminishing restraints under the NVT/NPT ensemble. All MD production runs were conducted under the NPT ensemble for a 100 ns simulation time using the OPLS3e force field.^29,37^ Subsequently, the trajectories were analyzed using the Simulation Event Analysis app embedded in Desmond to compute the C-alpha RMSD trajectory plots.

### MM-GBSA Calculations

The frames composing the MD trajectory obtained for the protein–protein complexes were analyzed with the Prime MM-GBSA tool included in the Schrödinger suite, as reported in a previous work.^38^ In greater detail, starting after system stabilization, one in every 400 frames (one every 3.7 ns) was considered for a total of 27 frames for each simulation. The complexes were refined with Prime under the OPLS3e force field adopting the Variable Dielectric Surface Generalized Born (VSGB) continuum solvation model.^39^ The energies obtained for the complexes were automatically calculated on the basis of the energy terms and the equation systems reported in the following
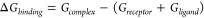

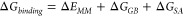

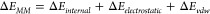
where Δ*G*_*binding*_ represents the total binding free energy upon protein–protein binding (LFA-1 considered as receptor and Orf7a considered as ligand); Δ*E*_*MM*_ is the total gas phase energy in the molecular mechanics (MM) force field (OPLS3e) and includes Δ*E*_*internal*_ arising from the bond, angle, and dihedral terms; Δ*E*_*electrostatic*_ and Δ*E*_*vdw*_ correspond, respectively, to the electrostatic and *van der Waals* energies; and Δ*G*_*GB*_ and Δ*G*_*SA*_ are the two solvation free energy contributions, respectively, the polar electrostatic solvation energy calculated via the generalized Born (GB) method and the nonelectrostatic solvation component (nonpolar contribution).^40,41^

The obtained MM-GBSA energy values were then averaged, and the standard deviation was calculated (Microsoft Corporation, Microsoft Excel 2018). Such values were also plotted over the simulation time to better visualize the stability of the system during the MD time frame.

### Clusterization and Superimposition with Efalizumab

The trajectories obtained by MD for the LFA-1/Orf7a models were clustered using the Schrödinger embedded application Desmond Trajectory Clustering. In particular, one in every 10 frames was considered, and the number of clusters was set to 3, obtaining three representative structures for each model. The efalizumab structure was retrieved from PDB (PDB ID: 3EOA) and prepared with the Schrödinger application Protein Preparation Wizard. The structure was then aligned with the three obtained by clusterization. The LFA-1 substructure belonging to the 3EOA model was removed, and three merged complexes were created. The *van der Waals* clashes between efalizumab and the computed models were counted using the Schrödinger Protein Interaction Analysis embedded application.

## Results and Discussion

### Generation of LFA-1/Orf7a Complex Models

Preliminary studies on the LFA-1/Orf7a complex previously appeared for SARS-CoV. In particular, a model was obtained by structure alignment of SARS-CoV Orf7a (PDB ID: 1YO4)^1^ with ICAM-1 present as a MIDAS ligand of LFA-1 in a reported complex (PDB ID: 1MQ8).^9^ In this model, Orf7a Glu26 was manually directed toward the magnesium ion by the authors, becoming part of its coordination sphere.^1^

In the current work, we initially adopted a similar approach to reproduce such a complex bound through MIDAS for LFA-1 and the SARS-CoV-2 Orf7a protein. In this connection, our sequence comparison studies for SARS-CoV and SARS-CoV-2 Orf7a showed over 90% identity, while the superimposition of their 3D structures provided an RMSD value of 0.94 Å and an Alignment Score of 0.035. Moreover, as reported by Nizamudeen et al., the LFA-1 binding determinant residues of SARS-CoV Orf7a, including Glu26, are maintained in the SARS-CoV-2 isoform.^11^ Briefly, the Orf7a structure (PDB ID: 6W37)^23^ was aligned using the Schrödinger 2020 application Protein Structure Alignment to the MIDAS ligands ICAM-1 and ICAM-3 present in two different complexes (PDB IDs: 1MQ8, 1T0P).^9,25^ The obtained structures resulted in being highly similar, with an RMSD between the two LFA-1/Orf7a complexes of 1.54 Å and an Alignment Score of 0.095. This prompted us to proceed with the highest resolution LFA-1 structure, namely 1T0P (1.66 Å).^25^ The resulting LFA-1/Orf7a complex was subjected to the already described protein preparation protocol, which comprehended a final restrained minimization step (OPLS3e). The carboxylate moiety of Glu26 resulted in a novel ionic interaction with the magnesium atom of MIDAS (3.5 Å). The obtained complex was named model0.

In this study, in order to explore the additional possible binding modes of SARS-CoV-2 Orf7a, a series of additional computational experiments were set up enrolling a protein–protein docking between LFA-1 and the SARS-CoV-2 Orf7a. For LFA-1, the X-ray structure of the i-domain at 1.7 Å resolution was set as the receptor (PDB ID: 3F74),^24^ while the SARS-CoV-2 Orf7a X-ray crystal structure at 2.9 Å was selected as the ligand (PDB ID: 6W37).^23^ The LFA-1 and Orf7a proteins were prepared with Schrödinger 2020 using the Protein Preparation Wizard following the procedure reported in the Materials and Methods section. The protein structures were uploaded in PDB format to the HDOCK server, and the docking process was submitted using default settings. The docking results and the PDB structures of the complexes for the 10 top ranked docking poses were then retrieved and analyzed (Table 1).

**Table 1:** Docking Scores for the Top 10 Best Poses of 3F74/6W37 Docking^a^

**model**	model1	model2	model3	model4	model5	model6	model7	model8	model9	model10
**score**	–189.28	–184.10	–182.58	–177.74	- 174.48	–173.76	–172.57	–172.48	–170.21	–168.19

a Values are expressed in kcal/mol.

MIDAS was previously reported to be directly interested in ICAM recognition,^42^ but it was not involved in most of the docked models obtained in this work. Nevertheless, it must be anyway considered that other binding motifs were described in the literature for some LFA-1 interactors. More specifically, efalizumab (PDB ID: 3EOA)^43^ and rhodocetin bind LFA-1 in a MIDAS-independent manner.^44^ Moreover, Jokinen et al. showed that human echovirus 1 (EV1) approaches integrin avoiding interaction with the metal ion region.^45^ Thus, other binding motifs cannot be ruled out. Following the docking experiment, the computed complexes were inspected to discard those bearing the β-propeller domain in a position which is not naturally available for interactions in LFA-1. This led to the exclusion of model3 and model5 from the set.

**Figure 1:**
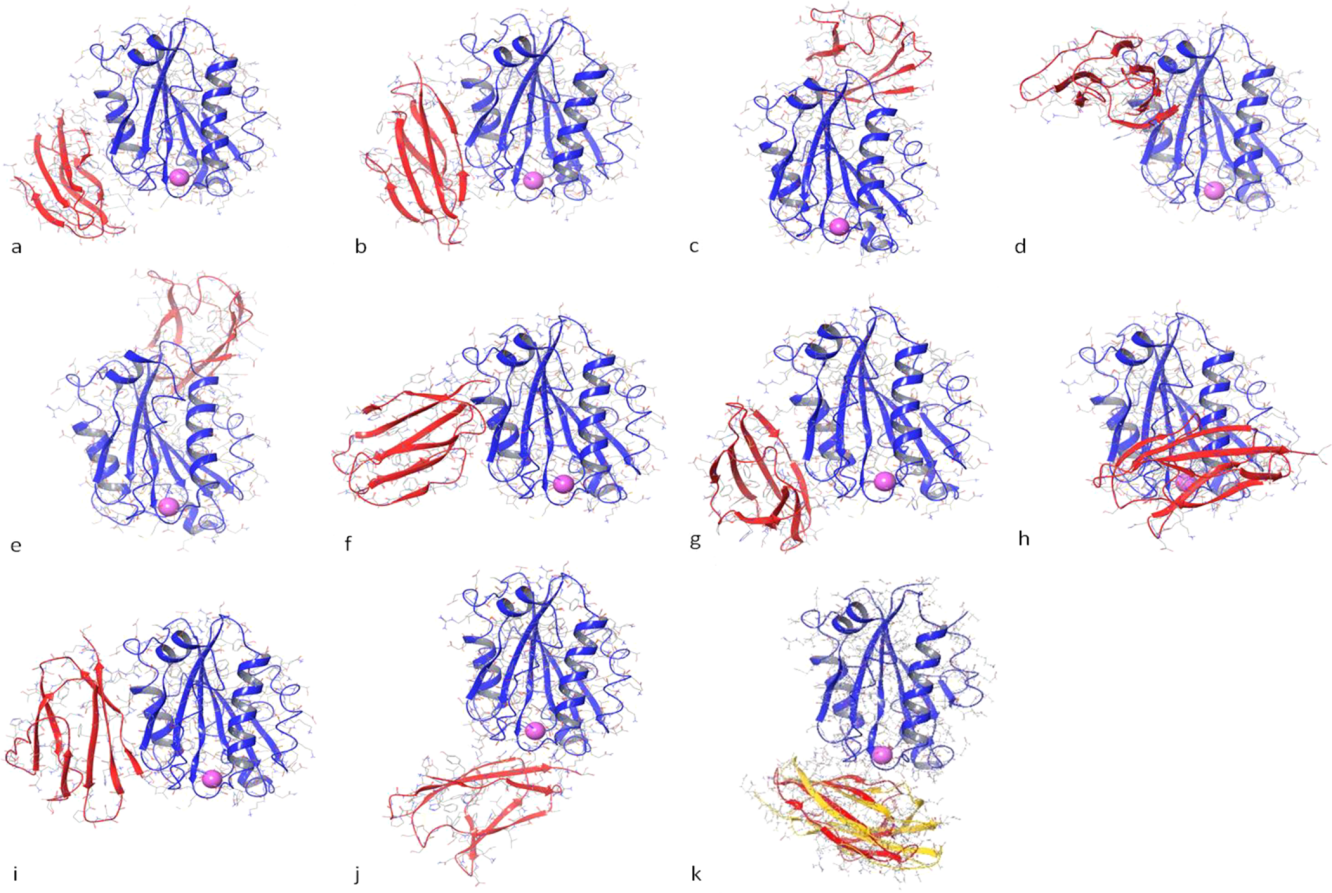
Overall representation of the protein–protein complexes obtained through docking studies and by protein structure alignment (a: model1, b: model2, c: model3, d: model4, e: model5, f: model6, g: model7, h: model8, i: model9, j: model10, k: model0 superimposed to ICAM-3). LFA-1 is represented in blue, SARS-CoV-2 Orf7a is represented in red, and ICAM-3 is represented in yellow. The magnesium ion is depicted in pink.

The whole set of protein–protein complexes resulting from structure alignment and docking studies (models), and that were then further investigated by MD simulations as will be described in the following, is reported in Figure 1.

### MD Simulations

**Figure 2:**
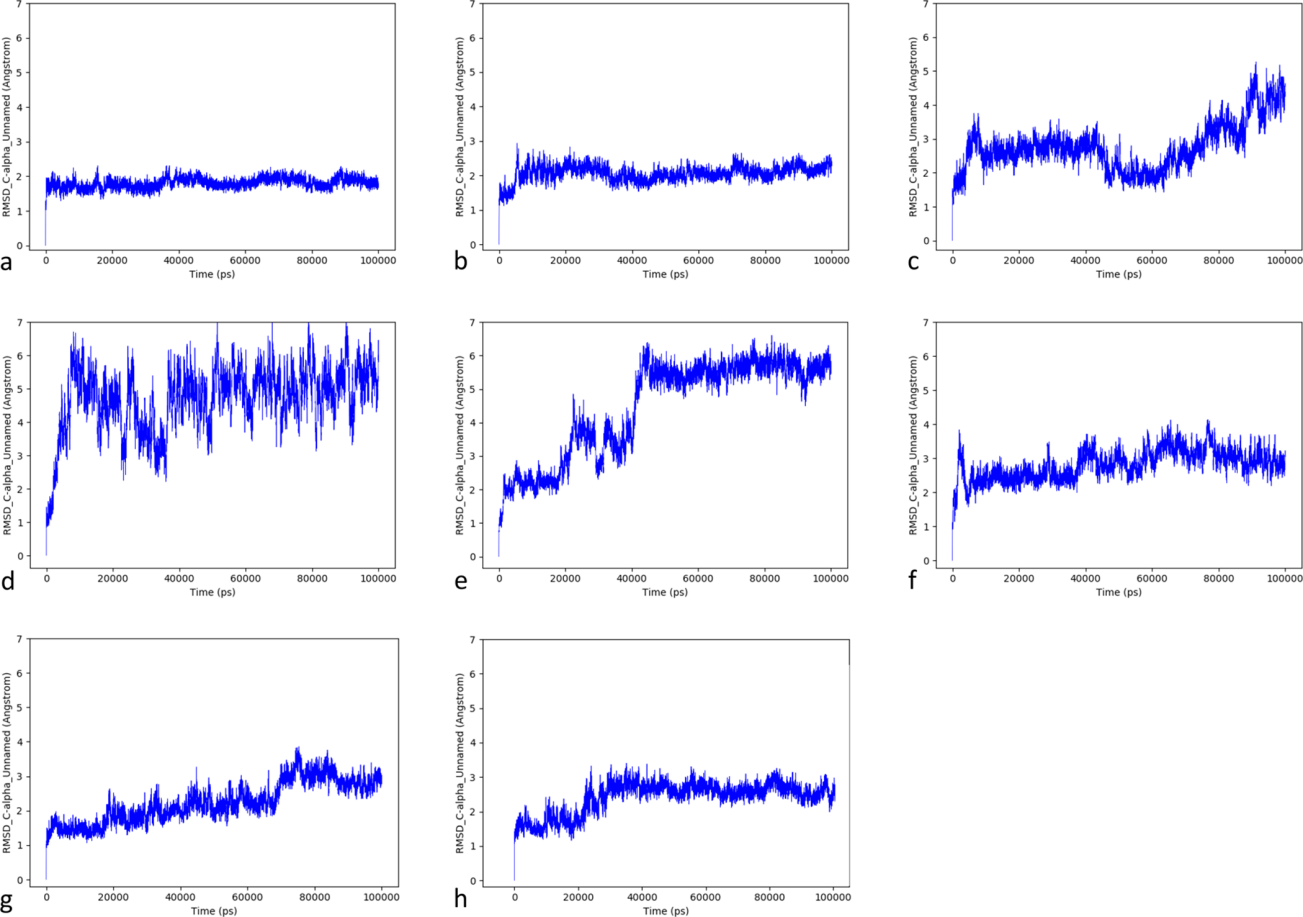
C-alpha RMSD (Å) trajectories over the MD simulation time for the LFA-1/Orf7a models (a: model1, b: model2, c: model4, d: model6, e: model7, f: model9, g: model10, h: model0). For comparison, the graphs were scaled to the same maximum RMSD value of 7.0 Å.

The set of models obtained by protein–protein docking (model1, -2, -4, -6, -7, -8, -9, -10) and by structure alignment (model0) was imported in Schrödinger 2020, and each complex was prepared with the Protein Preparation Wizard application, using default settings and with a final minimization step under the OPLS3e force field.^29^ In order to perform MD calculations for all these complexes, the systems were prepared with the System Builder application embedded in Desmond as previously described, and production runs were then performed under the OPLS3e force field for all models. The C-alpha RMSD values over simulation time were then analyzed for all the MD trajectories using the Simulation Event Analysis app embedded in Desmond 2020 (Figure 2).

According to the observed results, three models out of the nine considered demonstrated good stability in terms of C-alpha RMSD fluctuations over time (model1, model2, and model0), whereas models4, -6, -7, -8, -9, and -10 did not reach stabilization in the simulation time frame. However, model7 and model9 showed a tendency to reach stabilization even if higher fluctuations were registered. Model1 resulted in being the most stable complex, promptly reaching the stability at 1.8 Å RMSD and maintaining it throughout the simulation time frame with minor RMSD fluctuations. Model2 reached stability after 10 ns, showing slightly lower but satisfying stability. Model0, in which the interaction occurs through MIDAS, reached stability in 30 ns. This is probably due to the fact that, even if the resulting complex was subjected to a minimization step under the same MD force field, it was produced by rigid structure alignment, and it may require more time for equilibration. Additionally, as expected and as previously reported for the model with SARS-CoV Orf7a, the ionic interaction of Glu26 with the magnesium ion was maintained for all the simulation time.

### MM-GBSA Calculations

In order to determine the most reliable model among the stable ones, a free energy calculation study based on the MM-GBSA approach was performed on the simulation frames. MM-GBSA is a protocol that allows the binding free energy calculation between a receptor and a ligand based on different energy terms arising from the binding. The GB and SA energy terms are computed as the difference in solvent (water) interaction energy with the free receptor and free ligand and with the complex. MM is calculated considering the molecular mechanics energy derived from the interaction between the receptor and the ligand under the considered force field. This approach has been proven to be efficient also for various protein–protein complexes.^46−49^

**Figure 3:**
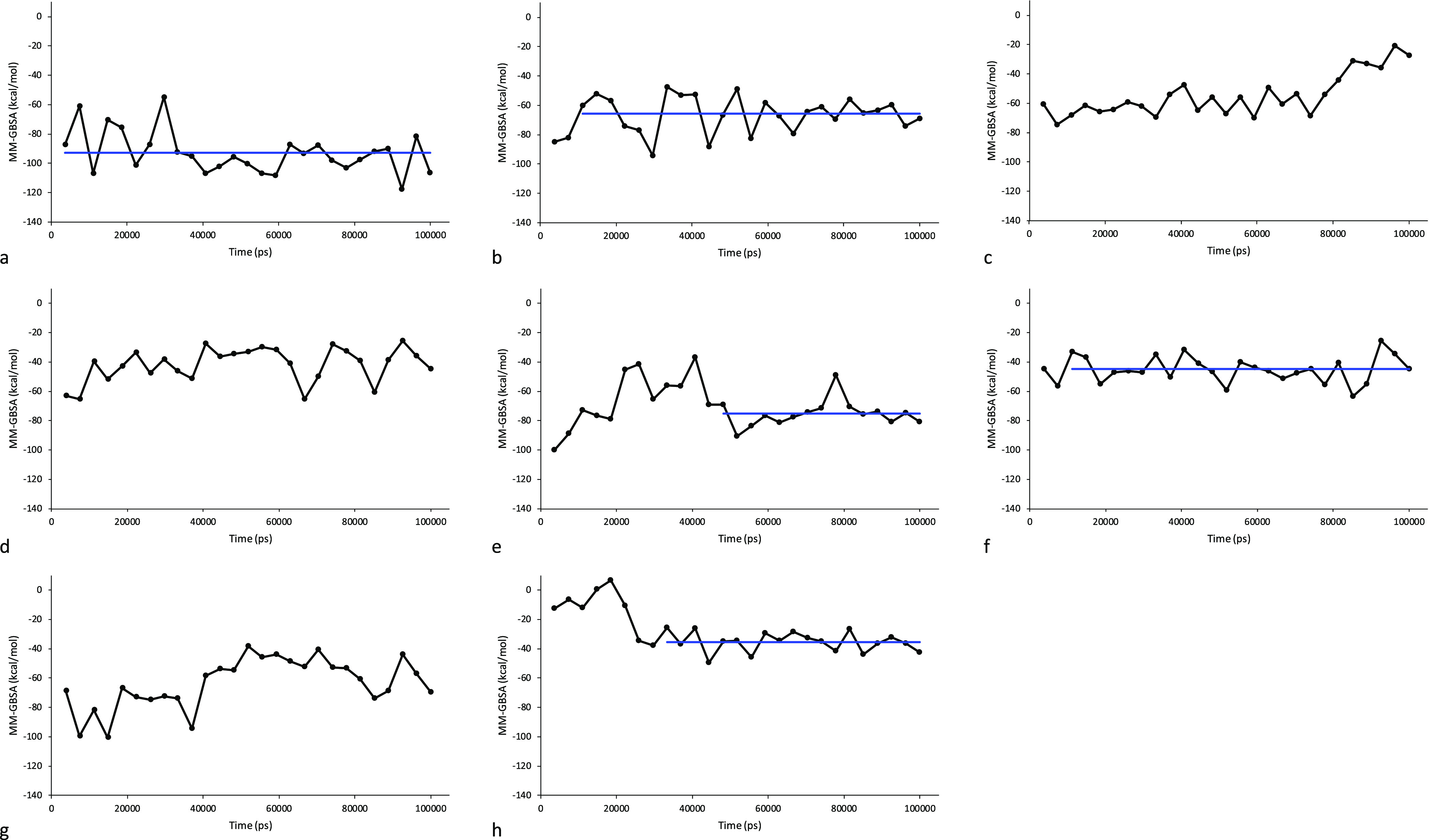
MM-GBSA (kcal/mol) graph over the MD simulation time for the LFA-1/Orf7a complex models. The averaged values are represented by a blue line (a: model1, b: model2, c: model4, d: model6, e: model7, f: model9, g: model10, and h: model0).

The calculations were performed using the Prime MM-GBSA application embedded in Schrödinger 2020, considering 3F74(24) as the receptor and 6W37(23) as the ligand. The VGBSA solvation model and the OPLS3e force field were used. The energy values were calculated for one in every 400 frames of the MD trajectory (for a total of 27 frames for each simulation). Such values are determined according to the RMSD values over time for the MD trajectories reported in Figure 2. The averaged values were calculated only if the system demonstrated stability in the simulation time frame; for this study, the complexes were considered stable when the fluctuations of RMSD were equal or lower than 1 Å for at least 50 ns.^50−52^ This was possible for model1, model2 (after 10 ns), model7 (after 45 ns), model9 (after 10 ns), and model0 (after 35 ns) but not for model4, model6, and model10. According to this analysis, model1 presented the lowest energy value of −92.8 ± 14.5 kcal/mol (Figure 3a) followed by model7 with −75.3 ± 9.1 kcal/mol (Figure 3e), model2 with −65.6 ± 12.1 kcal/mol (Figure 3b), model9 with −44.9 ± 9.1 kcal/mol (Figure 3f), and model0 with −35.4 ± 6.8 kcal/mol (Figure 3h). With respect to this, the MM-GBSA value for model0 was the most stable over simulation time.

### Comparison with the Efalizumab Binding Site

**Figure 4:**
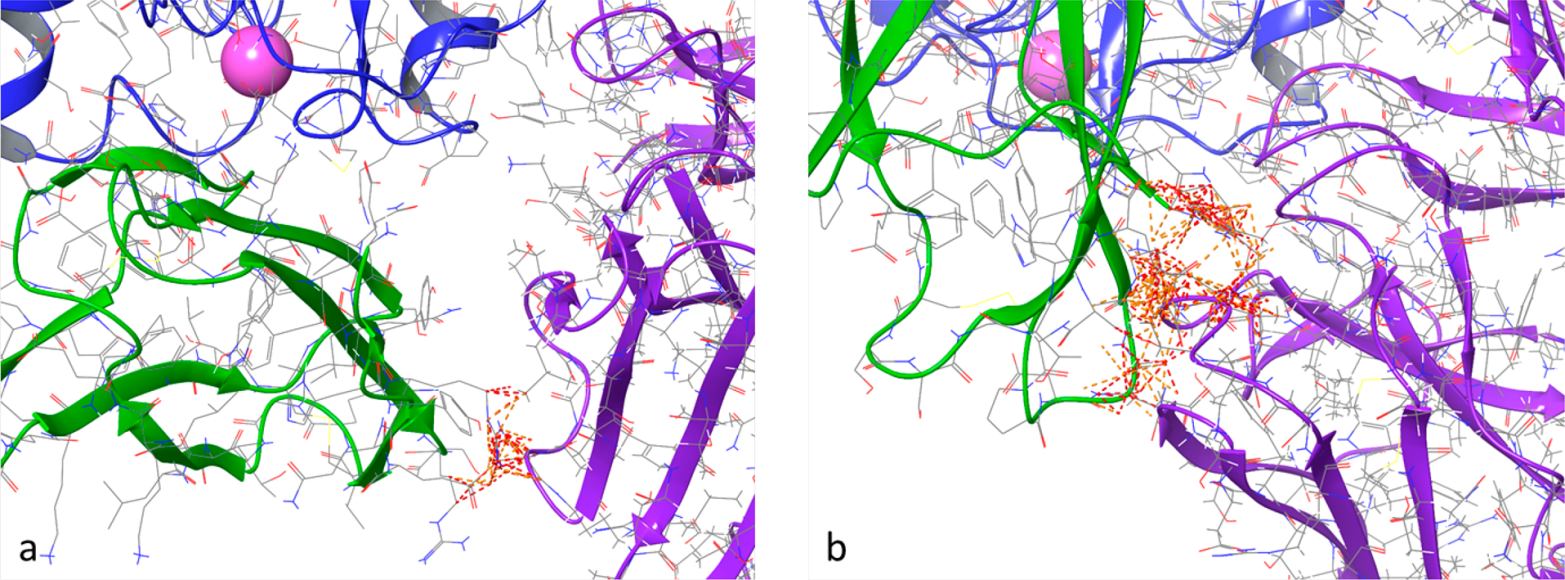
Superimposition of the efalizumab/LFA-1 complex with computed models involving Orf7a (a: model0, b: model9). The depicted structures are the ones showing the highest number of clashes with respect to the three obtained by clusterization for each model. The clashes are represented as dashed lines (in orange/red) between efalizumab (in purple) and Orf7a (in green). LFA-1 is depicted in blue.

As anticipated, efalizumab is reported to bind LFA-1 without interfering with MIDAS.^43^ Some of the computed models described above structurally resemble this interaction pattern. In order to study the presence of direct steric hindrance between the computed models and efalizumab, each MD trajectory obtained for the models generated by protein–protein docking (model1, model2, model7, model9) and model0 was cut based on the stability range (over time C-alpha RMSD) and clustered through the affinity propagation clustering method obtaining three representative structures. These were directly superimposed to the crystal structure of efalizumab in complex with LFA-1 (PDB ID: 3EOA).^43^ More specifically, model0 and model9 revealed a considerable amount of close contacts between the residues of the two respective substructures with a maximum number of 120 clashes for model0 and 603 for model9 (Figure 4).

## Conclusions

In our computational study, we aimed at providing structural insight into the putative LFA-1/Orf7a macromolecular assembly by means of protein–protein docking, MD simulations, MM-GBSA approach, and structure comparison. The computed models, resulting from docking studies or structure alignment and refined by the following experimental steps, suggest more than a possible binding motif between Orf7a and LFA-1. These observations support the data from the literature showing that LFA-1 interacts with its partners through MIDAS or in a metal ion-independent manner. The model bearing the MIDAS interaction pattern, obtained by structure alignment, demonstrated good stability in the MD simulation and satisfying values in the MM-GBSA experiment. Moreover, the ionic interaction of Glu26 with the metal, spontaneously generated during the minimization step of protein preparation, was retained throughout the entire simulation. Overall, the reliability of this model is also supported by previously reported *in vitro* binding data and structural clues on the LFA-1 and SARS-CoV Orf7a interaction, together with the similarity with the LFA-1/ICAM complex. Nevertheless, a different binding pattern, which has been previously described for other macromolecular partners of LFA-1, cannot be ruled out.

This work aims at triggering the interest of the scientific community toward SARS-CoV-2 Orf7a and its complex with LFA-1 as putative targets and to prompt the testing of the above-mentioned findings on *in vitro* models. In light of these results, the current study paves the way for the design and screening of small molecules interacting with the assembly and potentially interfering with virus–host interactions and pathogenicity.

## Notes

Data and Software Availability: For this study, HDOCK (available at http://hdock.phys.hust.edu.cn/) and Schrödinger Release 2020-1 (available at https://www.schrodinger.com/) were used. PDB structures are available from RCSB PDB (https://www.rcsb.org). Excel is available at the Microsoft Office Web site (https://www.microsoft.com/en-us/download/office.aspx). Produced and analyzed data are available from the authors upon request.
